# Are Advanced Glycation End-Products and Skin Autofluorescence Associated with E-Selectin and Pulse Wave Velocity as Markers of Atherosclerosis Risk in Children with Obesity?

**DOI:** 10.3390/ijms26209966

**Published:** 2025-10-13

**Authors:** Anna Medyńska, Anna Noczyńska, Danuta Zwolińska

**Affiliations:** 1 Department and Clinic of Pediatric Nephrology, Medical University of Wrocław, 50-367 Wrocław, Poland; danuta.zwolinska@umw.edu.pl; 2 Department and Clinic of Pediatrics, Endocrinology, Diabetology and Metabolic Diseases, Medical University of Wrocław, 50-367 Wrocław, Poland; anna.noczynska@umw.edu.pl

**Keywords:** children, obesity, atherosclerosis, advances glication end products (AGEs), skin autofluorescens (sAF)

## Abstract

Obesity is a risk factor for numerous complications, including atherosclerosis, the pathogenesis of which is complex. The aim of our study is to evaluate serum levels of E-selectin and hs-CRP and pulse wave velocity (PWV) as atherosclerosis risk factors and to explore their relationship with advanced glycation end products (AGEs), methylgioxal (MG) and skin autofluorescence (sAF). We evaluated 125 children aged 8–18 years with simple obesity, stratified into two subgroups based on SDS BMI (Group I: 2–4; Group II: >4), and compared them with 33 age-matched peers of normal weight. Children with obesity exhibited significantly elevated serum concentrations of AGEs, MG, E-selectin, and hs-CRP relative to the control group. Additionally, both height-normalized pulse wave velocity (SDS PWV) and sAF values were significantly higher in the children with obesity compared to their normal-weight counterparts. Except for sAF, which was elevated in children with obesity with a higher SDS BMI, no significant differences were observed among the subgroups of children. Positive correlations were observed between E-selectin and AGEs, MG and SDS PWV, as well as sAF and SDS BMI. Our findings indicate that children with obesity exhibit early signs of atherosclerotic changes, regardless of the degree of obesity. Moreover, circulating AGEs may represent a more reliable biomarker of atherosclerosis risk than sAF, as suggested by the strong positive correlation between AGEs and E-selectin. Further studies are warranted to validate these findings.

## 1. Introduction

Obesity is a chronic inflammatory disease with many complications that impair quality of life and increase the risk of morbidity and mortality [1]. Atherosclerosis and related cardiovascular disease are among the most prominent of these. It is well known that the risk of developing atherosclerosis affects not only adults with obesity, but children too. Years ago, autopsy studies from the Bogalusa Heart Study showed early atherosclerotic lesions in the form of fatty streaks in arteries, as well as increased thickness of the middle and internal carotid artery membrane (IMT), in adulthood [2]. The atherosclerotic process, which begins with endothelial damage, is complex. One of the mechanisms leading to microvascular and macrovascular damage is the excessive production of advanced glycation end products (AGEs). These are formed when sugars combine with proteins and lipids in a non-enzymatic process. Food is an exogenous source of AGEs, especially meat prepared at high temperatures, as well as fast food and highly processed food [3]. Obesity is often accompanied by hyperglycaemia, insulin resistance, oxidative stress and chronic inflammation, all of which enhance the formation of AGEs. The atherosclerotic activity of AGEs is multifactorial. They form permanent cross-links with collagen and elastin in the vessel wall. After binding to the RAGE (receptor for advanced glycation end-products) on endothelial cells, they enhance the production of free oxygen radicals (ROS) and activate the inflammatory process by producing pro-inflammatory cytokines (TNF-α and IL-6) and adhesion molecules (ICAM-1 and VCAM-1). These processes lead to further vascular damage and the formation of atherosclerotic plaque [4,5].

Methylglyoxal (MG) is one of the precursors of AGEs. It plays an important role in the initiation of atherosclerotic lesions by reducing nitric oxide (NO) production, which leads to endothelial dysfunction. In adults under hyperglycaemic conditions, MG is considered an indicator of endothelial damage, vascular stiffness, and increased blood pressure [6]. Circulating AGEs are usually evaluated through the analysis of individual AGE compounds. The tissue accumulation of AGEs can also be examined using the AGE-Reader, which employs the skin autofluorescence (sAF) phenomenon [7,8]. Unlike adults, there are very few studies on AGEs and sAF in children with obesity, and none at all regarding the development of early atherosclerotic lesions.

Therefore, the aim of our study is to evaluate serum levels of E-selectin and hs-CRP and PWV as atherosclerotic risk factors and to explore their relationship with AGEs, MG and sAF.

## 2. Results

There were no significant differences in age or gender between the children with obesity and the healthy controls. However, BMI, SDS-BMI, SBP, and DBP were higher in the children living with obesity compared to the controls. Detailed data are shown in Table 1. Children with obesity had significantly higher mean serum triglyceride levels and lower HDL cholesterol levels than their lean counterparts. Although total and LDL cholesterol levels were slightly higher in the group with obesity, the differences were not statistically significant. Mean fasting glucose levels were also significantly elevated in children with obesity. Nonetheless, only four individuals in this group had values above the normal range, and these same patients also showed abnormal results after 120 min of the glucose load test. Detailed data are provided in Table 2. Comparing children with obesity according to SDS BMI showed that patients with SDS BMI > 4 had only higher values of body weight and diastolic blood pressure (Table S1, Supplementary Files). In children with obesity, higher serum concentrations of AGEs, MG, E-selectin, and hs-CRP were observed compared to the control group. Similarly, SDS PWV values normalized for height were higher in children with obesity than in their healthy peers, which is consistent with findings reported by other authors [9,10,11]. Likewise, the sAF values of children with obesity were significantly higher compared to those of controls. Detailed data are shown in Table 2. There were no statistically significant differences in the above parameters among children according to their BMI values (Table S2, Supplementary Files).

Table S3 (Supplementary Files) presents a summary of lipid and carbohydrate metabolism disorders in the studied population of children with obesity.

**Figure 1:**
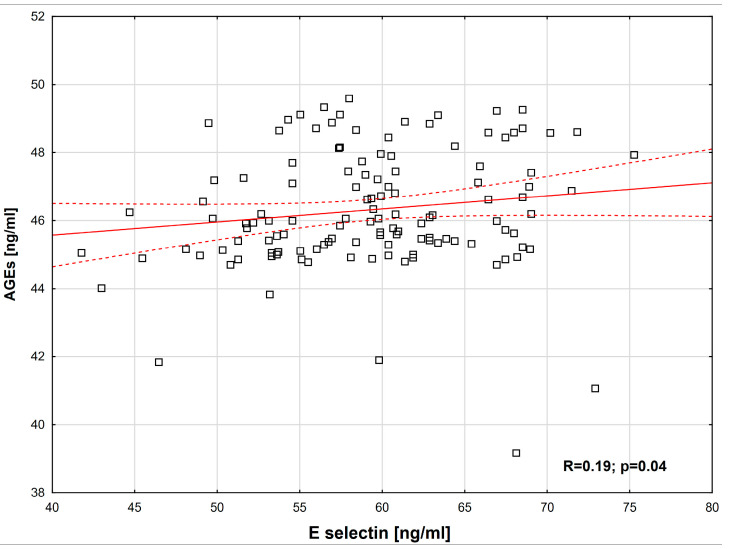
AGEs correlation depending on E-selectin (Spearman’s correlation coefficient, R = 0.19, *p* = 0.04).

**Figure 2:**
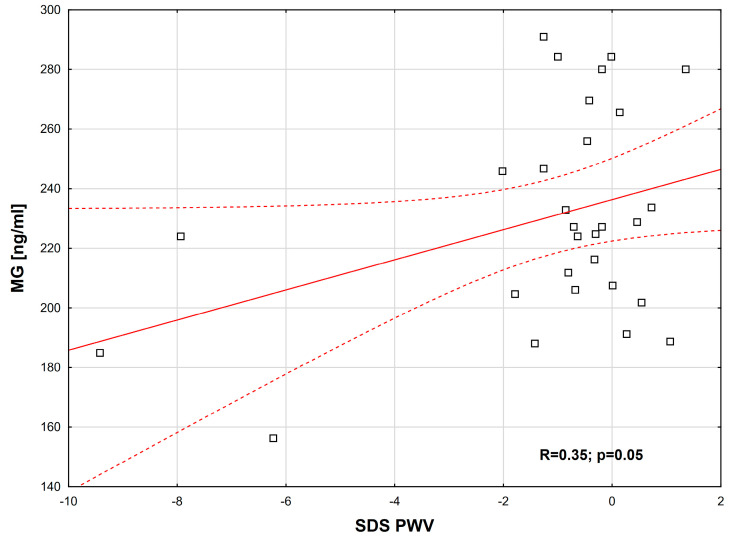
MG correlation depending on SDS-PWV (Spearman’s correlation coefficient, R = 0.35, *p* = 0.05).

**Figure 3:**
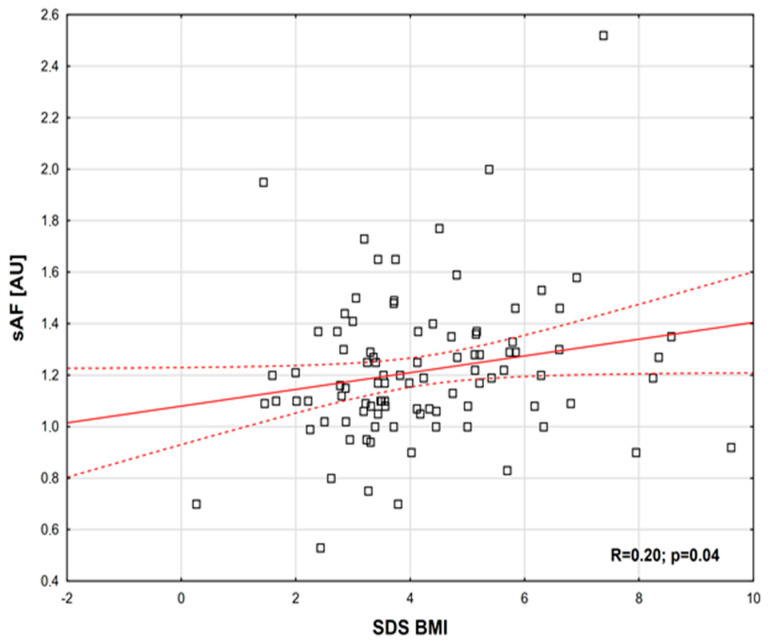
sAF correlation depending on SDS BMI (Spearman’s correlation coefficient, R = 0.20, *p* = 0.04).

The following positive correlations were observed: between E-selectin and AGEs (R = 0.19, *p* = 0.04) (Figure 1); between MG and SDS PWV (normalized for height) (R = 0.35, *p* = 0.05) (Figure 2); between sAF and SDS BMI (R = 0.20, *p* = 0.04) (Figure 3). Additionally, a borderline positive correlation was found between sAF and hs-CRP (R = 0.18, *p* = 0.06). Although not statistically significant, a trend toward a positive correlation was noted between AGEs and MG (R = 0.23, *p* = 0.08).

No statistically significant associations were identified in the multiple regression models. Detailed data are presented in Table S4 (Supplementary Files).

In contrast to some authors [12,13], and consistent with the findings of Manco et al. [14], we did not observe any correlation between E-selectin and lipid metabolism parameters. Likewise, in agreement with Hudson et al. [10], no associations were found with the degree of obesity, arterial hypertension, or metabolic abnormalities.

## 3. Discussion

Obesity is widely recognized as a state of low-grade inflammation that leads to endothelial damage, arterial stiffness, and accelerated atherosclerosis. AGEs are implicated in this process. Nevertheless, their relationship with vascular dysfunction in children with obesity remains unexplored, both in relation to circulating AGEs and to those accumulated in tissues.

We observed significantly higher serum AGE levels in children with obesity compared to their normal-weight peers, particularly for methylglyoxal, a key AGE precursor not detected by the commercial assay employed. Consistent with our findings, Uribarri et al. reported similar results in adults with obesity and metabolic abnormalities, whereas individuals without such conditions did not exhibit elevated MG or Nε-carboxymethyllysine (CML) levels [15]. In our cohort, metabolic disturbances were present in 73.6% of patients, which may account for the observed increases in AGE levels.

Uribarri et al. reported associations between AGE levels and inflammatory markers but found no correlation with BMI or adipose tissue content [15]. In line with these findings, our study also identified no relationship between AGE concentrations and either BMI or SDS-BMI subgroups. Elevated AGE levels may result from increased dietary intake, augmented endogenous production, or impaired clearance from tissues.

While studies in adults have linked high dietary AGEs intake to higher BMI, they often did not assess circulating AGE levels [16]. The only study investigating this relationship in children with obesity confirmed these findings [17]. Coppola et al. further demonstrated that obese individuals consumed more ultra-processed foods (UPFs) and dietary AGEs than their normal-weight peers [17]. Given that UPFs are rich in AGEs, this may partly explain our observations. Moreover, a trial by Vlassara et al. in adults showed that reducing dietary AGEs intake decreased circulating AGE levels, inflammation, and oxidative stress [18].

However, studies on circulating CML in adults with obesity have provided inconsistent results, with reports of both increased [19,20] and decreased levels [21,22]. Data in children are scarce. Garay-Sevilla et al. found no difference in serum CML between obese and normal-weight metabolically healthy children [23], whereas Sebekova et al. observed lower AGE levels, including CML, fructosyl-lysine, and AGE-associated fluorescence, in children with obesity [24]. It should be emphasized, however, that their study included only 18 participants aged 5–15 years [24]. This may have influenced the results, as previous studies have shown that younger children consume substantially fewer ultra-processed foods, and serum CML levels correlate with age [5].

Sebekova et al. also associated reduced AGE levels with glomerular hyperfiltration. However, assessing kidney function in children with obesity is challenging, as excess adiposity may lead to overestimation of eGFR, thereby potentially masking underlying kidney injury. In our cohort, eGFR suggested hyperfiltration, yet we also observed evidence of subclinical nephron injury, including elevated C-terminal fragment of agrin (tCAF), a proposed marker of renal injury [25].

Finally, methodological differences may account for discrepancies across studies. It is well established that CML preferentially accumulates in adipose tissue, thereby influencing serum concentrations. Accacha et al. confirmed this observation in a cohort of 88 children, 44% of whom were overweight or obese [5]. In our study, we employed an ELISA kit (Wuhan EIAab Science) to quantify total AGE levels; however, this assay does not distinguish between individual AGE species such as CML or pentosidine, instead providing a cumulative measure of AGEs.

To our knowledge, this is the first study in children to demonstrate an association between AGE levels and vascular dysfunction. We observed a positive correlation between serum AGE concentrations and E-selectin, as well as between MG and SDS-PWV. Furthermore, sAF was significantly elevated in children with obesity, particularly in the most severely affected group, and sAF showed a positive correlation with obesity severity.

These results are consistent with previous findings by Lentferink et al., who reported no association between sAF and age or components of the metabolic syndrome [26]. Similarly, Coppola et al. observed elevated sAF levels in children with obesity, accompanied by mitochondrial alterations in peripheral blood mononuclear cells (PBMCs), which may contribute to increased production of reactive oxygen species and vascular endothelial injury [17].

While sAF is commonly elevated in adults with obesity [27,28,29], its relationship with metabolic status remains inconclusive. Some studies, including Apaydin et al., reported no differences according to metabolic health [30], whereas others observed significant associations [28,31]. Notably, higher sAF levels have been reported in individuals with increased waist circumference, suggesting that visceral adiposity may play a key role in AGE accumulation.

In our study, no direct correlation was observed between serum AGE levels, MG, and skin autofluorescence. A possible explanation is that circulating AGEs are dynamic, with concentrations influenced by diet, glycaemic control, inflammatory status, and renal clearance. In contrast, tissue AGEs are more stable and cumulative, reflecting long-term exposure to hyperglycaemia, oxidative stress, and aging.

Skin autofluorescence reflects AGEs associated with long-lived tissue proteins, such as collagen, and therefore does not necessarily correspond to the instantaneous serum AGE concentrations. Additionally, not all AGEs are fluorescent (e.g., methylglyoxal), making them undetectable by the device due to differences in fluorescence properties among individual AGE species. In our study, except for a weak correlation between sAF and hs-CRP, a known risk factor for atherosclerosis, no correlations were observed with the early atherosclerosis markers assessed.

## 4. Materials and Methods

### 4.1. Study Overview

This is part of a large-scale project carried out on a group of paediatric patients with obesity, with the aim of comprehensively assessing renal function and the development of premature atherosclerosis.

### 4.2. Subjects

This prospective cohort study group consisted of 125 children with simple obesity (68 girls and 57 boys) aged 8–18 years and recruited from the Clinic of Pediatrics, Endocrinology, Diabetology, and Metabolic Diseases at the Medical University of Wrocław, Poland. Children with secondary obesity, acute or chronic infections and undergoing immunomodulatory treatment were excluded. According to their body mass index standard deviation score (SDS BMI), the patients were divided into two subgroups: I—comprised 65 children (SDS BMI > 2 < 4) and II—consisted of 60 individuals (SDS BMI > 4). 33 healthy, age-matched peers with normal body weight served as the control group.

Informed consent was obtained from parents, and from children over 16 years of age. Ethics approval was granted by the Ethics Board of Wrocław Medical University (No. 376/2016; 12 July 2016) and conducted in accordance with the Declaration of Helsinki.

Each participant underwent a comprehensive medical interview and physical examination. Standing height was recorded to the nearest 0.1 cm using a Harpendensta-diometer, while body weight was measured with an electronic scale (SECA, Hamburg, Germany) to the nearest 0.05 kg. BMI was calculated by dividing weight (kg) by height squared (m^2^) and expressed as a standard deviation score (SDS), adjusted for age and sex according to national percentile charts [32]. Obesity was defined as a BMI greater than 2 SDS, following WHO guidelines.

Blood pressure (BP) was measured using the oscillometric method (Omron 705 IT, Omron Healthcare Co., Kyoto, Japan) with appropriately sized cuffs under standard conditions. According to European guidelines [33], hypertension in children up to 15 years of age was defined as systolic and/or diastolic BP values that met or exceeded the 95th percentile for sex, age, and height (based on Polish reference charts from the OLAF study [34]). In individuals aged 16 years and older, hypertension was diagnosed if BP exceeded 140/90 mmHg in at least three separate readings.

Venous blood samples were collected in the morning in a fasting state, centrifuged at 3000 rpm for 15 min, and the serum was stored at −80 °C until analysis for total cholesterol, HDL, LDL, triglycerides, fasting glucose, insulin and creatinine in all subjects. The Homeostatic Model Assessment of Insulin Resistance (HOMA-IR) was calculated using the following formula:

HOMA-IR = Fasting glucose (mg/dL) × Fasting insulin (μU/mL)] / 405(1)

Insulin resistance was diagnosed when the HOMA-IR value exceeded 4.4 in children over 12 years of age [35] and 3.16 in younger children [36]. Estimated glomerular filtration rate (eGFR) was also calculated using the Schwartz formula [37]. Furthermore, serum levels of AGEs, MG, E-selectin, and hs-CRP were measured in all subjects. AGEs were assessed using an ELISA kit (Wuhan EIAab Science Co., Ltd., Wuhan, China, catalog number E0263h), with a method sensitivity of 0.41 ng/mL.

MG was quantified using a commercially available ELISA kit (Wuhan Fine Biotech Co., East Lake High-tech development Zone, Wuhan, China, catalog number EH4248), with a method sensitivity < 9375 ng/mL.

Serum hs-CRP was quantified using a commercially available ELISA kit (Demeditec Diagnostics GmbH, Kiel, Germany; catalog number DE740011), with a method sensitivity of 0.02 μg/mL.

E-selectin level was measured with an ELISA kit from R@D System, INC. Minneapolis, MN, USA, catalog number DSLE00, with a method sensitivity of 0.009 ng/mL.

### 4.3. Skin Autofluorescence Measurements

Skin autofluorescence measurements were performed using the AGE Reader system (Diagnoptics BV, Groningen, The Netherlands) at room temperature. For each participant, the measurement was taken on the medial surface of the right forearm. The device emits light waves with a wavelength of 300–420 nm. The light passes through a 4 cm^2^ window and directly illuminates the skin. A spectrometer integrated into the device (Avantes Inc., Eerbeek, The Netherlands) records the reflected and emitted light from the skin in the range of 300–600 nm. Each patient underwent the measurement three times, and the final result was the average of all three readings. The sAF value was expressed in arbitrary units (AU).

### 4.4. Pulse Wave Velocity (PWV) Measurement

First, the children’s arterial BP was measured. Next, while the child was lying supine, the measurement points for PWV were marked over the carotid and femoral arteries. The straight-line distances from these points to the sternal notch were then measured. PWV was assessed using a tonometric transducer (Miller Instruments, Inc., Houston, TX, USA) connected to a SphygmoCor recording device (AtCor Medical Pty. Ltd., Sydney, Australia) and a computer equipped with the corresponding signal analysis software. To obtain the signal, the tonometer was placed directly on the skin over the artery. Once the software confirmed a high-quality signal, pulse wave data were recorded for approximately 30 s. The first measurement, taken over the internal carotid artery, represented the proximal site (closer to the heart), while the second, taken over the femoral artery, represented the distal site. The procedure was repeated three times, and the average value was calculated. PWV was calculated using the formula PWV = ds/dt, where ds is the distance difference between the femoral and carotid measurement sites from the sternal notch (measured in meters), and dt is the time interval between successive R-waves on the ECG and the onset of the corresponding pulse waves (measured in seconds). To account for the effect of height on pulse wave measurements, SDS-PWV was computed. All measurements were conducted in person. The characteristics of the study groups are presented in Table 1 and Table 2.

### 4.5. Statistical Analysis

For all groups, descriptive statistics of the continuous variables included the mean (x), median (M), range (min–max), interquartile range (25th–75th percentile), and standard deviation (SD). Differences in mean values between independent groups were assessed using the non-parametric Mann–Whitney U test, after verifying homogeneity of variances with the Bartlett test. Linear regression analysis was used to evaluate selected parameter pairs, with Pearson’s correlation coefficient calculated to assess the strength of relationships. A *p*-value of ≤0.05 was considered statistically significant. Statistical analyses were carried out using the EPIINFO software, version 7.1.1.14 (release date: 2 July 2013).

The effects of AGEs, MG, and sAF on E-selectin, hsCRP, and SD_PWV_height were analyzed using a multiple regression model, with sex, age, height percentile, and BMI percentile included as covariates. Model adequacy was assessed using the variance inflation factor (VIF) to check for multicollinearity among predictors, the Shapiro–Wilk test to assess the normality of residuals, and the Breusch-Pagan test to evaluate homoscedasticity of residuals. When the assumption of normally distributed residuals was not met, quantile regression (for the median) was applied; otherwise, ordinary least squares (OLS) regression was used. All analyses were conducted in R version 4.4.2 using the packages ‘stats 4.1.2’ (OLS regression, Shapiro–Wilk test), ‘quantreg 5.99’ (quantile regression), ‘car 3.1-3’ (VIF calculation), and ‘lmtest 0.9-40’ (Breusch-Pagan test).

## 5. Summary

Children with obesity exhibit features of atherosclerosis risk, as indicated by elevated serum E-selectin levels and increased arterial stiffness measured by height-adjusted pulse wave velocity. Additionally, these parameters were not affected by age, gender, or BMI. Our findings demonstrate a significant association between circulating AGE levels and E-selectin, as well as between MG and SDS-PWV. These results suggest that circulating AGEs may play a key role in the pathogenesis of atherosclerosis in children with obesity and could serve as a more sensitive marker of early atherosclerotic changes than skin autofluorescence.

## Figures and Tables

**Table 1:** Characteristics of the studied groups.

Variable		Control Group F/M 18/15	Children with Obesity F/M 68/57	*p*
Age [years]	mean ± SD	12.9 ± 3.0	13.7 ± 2.84	0.172
	range (min–max)	7.6–17.8	8.0–17.9	
Body weight [kg]	range (min–max)	47.7 ± 11.9	85.7 ± 23.8	0.0001 *
	median	48.1	82.7	
	quartile (25–75Q)	38.6–55.4	72.7–100	
BMI	range (min–max)	19.2 ± 2.3	32.1 ± 5.8	0.0001 *
	median	19	30.8	
	quartile (25–75Q)	17.7–20.3	28.4–35.2	
SDS BMI	range (min–max)	0.061 ± 0.633	4.02 ± 1.7	0.0001 *
	median	0.061	3.55	
	quartile (25–75Q)	(−0.547)–0.563	2.88–5.13	
SBP [mmHg]	mean ± SD	106.3 ± 8.9	117.2 ± 9.9	0.0001
	range (min–max)	85–120	98–140	
DBP [mmHg]	mean ± SD	65.3 ± 7.2	71.8 ± 8.0	0.0001
	range (min–max)	48–76	50–92	
Total cholesterol [mg/dL]	mean ± SD	164.1 ± 14.8	179.2 ± 134.9	0.537
	range (min–max)	133–188	111–1611	
HDL-cholesterol [mg/dL]	mean ± SD	59 ± 9.6	42.2 ± 8.4	0.0001
	range (min–max)	34–78	27–65	
LDL-cholesterol [mg/dL]	mean ± SD	94.1 ± 14.9	100.4 ± 24.4	0.175
	range (min–max)	65–121	49–184	
Triglycerides [mg/dL]	range (min–max)	57–120	39–469	0.0021 *
	median	94	107	
	quartile (25–75Q)	74–105	83.5–141	
Creatinine [mg/dL]	mean ± SD	0.736 ± 0.144	0.629 ± 0.123	0.0001
	range (min–max)	0.54–1.19	0.37–0.89	
eGFR [mL/min/1.73 m^2^]	mean ± SD	125 ± 13.2	154 ± 25.1	0
	range (min–max)	96.0–160.0	109–235	
Fasting glucose [mg/dL]	range (min–max)	75–94	56–153	0.0118 *
	median	88	82	
	quartile (25–75Q)	85–91	77–82	

* analysis with the non-parametric Mann–Whitney U test.

**Table 2:** sAF, PWV and serum E-selectin, AGEs, MG and hsCRP in the studied groups.

		Control Group	Children with Obesity	*p*
E-selectin [ng/mL]	range (min–max)	22.3–37.4	41.8–75.3	0.0001 *
	median	31.5	59.4	
	quartile (25–75Q)	29.4–34.9	54.6–63.4	
AGEs [ng/mL]	range (min–max)	22.1–26	39.2–49.6	0.0001 *
	median	24.3	46	
	quartile (25–75Q)	23.8–25.1	45.2–47.5	
MG [ng/mL]	range (min–max)	41.2–85.9	156.4–302.5	0.0001 *
	median	69.3	227.2	
	quartile (25–75Q)	50.7–78.7	211.9–253.1	
hs-CRP [µg/mL]	range (min–max)	1.02–1.45	2.36–4.46	0.0001 *
	median	1.2	3.22	
	quartile (25–75Q)	1.14–1.32	2.86–3.54	
sAF [AU]	mean ± SD	1.02 ± 0.23	1.21 ± 0.29	0.00072
	range (min–max)	0.7–1.7	0.53–2.52	
SDS PWV	mean ± SD	−1.54 ± 1.46	–0.55 ±1.97	0.0106
	range (min–max)	(−4.08)–0.97	(−9.43)–2.33	

* analysis with the non-parametric Mann–Whitney U test.

## Data Availability

The original contributions presented in this study are included in the article and Supplementary Material. Further inquiries can be directed to the corresponding author.

## Supplementary Materials

The following supporting information can be downloaded at: https://www.mdpi.com/article/10.3390/ijms26209966/s1.
