# Supplementary material for: Are Advanced Glycation End-Products and Skin Autofluorescence Associated with E-Selectin and Pulse Wave Velocity as Markers of Atherosclerosis Risk in Children with Obesity?
Source: Int J Mol Sci. 2025 Oct 13;26(20):9966. doi: 10.3390/ijms26209966 (PMC12563969; doi:10.3390/ijms26209966)
Supplement: Supplementary file 1 [file ijms-26-09966-s001.zip › ijms-3798322-supplementary.pdf]

Table S1. Patients' characteristics and selected biochemical parameters according to the SDS BMI values.

Variable		2 ≤ SDS BMI ≤ 4<br>n=65 F/M 32/33	SDS BMI > 4<br>n = 60 F/M 36/24	p
Age [years]	mean ± SD<br>range (min-max)	13.7 ± 2.8<br>8 – 17.9	13.5 ± 2.9<br>8.2 – 17.8	0.729
Body weight [kg]	range (min –max)<br>median<br>quartile (25–75Q)	34 – 112.2<br>78.2<br>70.5 – 88.4	38 – 170.9<br>99.3<br>79.9 – 113.3	0.0001*
SBP [mmHg]	mean ± SD<br>range (min-max)	116.1 ± 9.4<br>98 – 140	119.3 ± 10.4<br>99 – 140	0.0959
DBP [mmHg]	mean ± SD<br>range (min-max)	70.3 ± 7.7<br>50 – 90	73.9 ± 7.9<br>59 – 92	0.0172
Total cholesterol [mg/dl]	mean ± SD<br>range (min-max)	170.5 ± 30.9<br>121 – 259	193 ± 208.8<br>111 – 1611	0.414
HDL-cholesterol [mg/dl]	mean ± SD<br>range (min-max)	42.8 ± 8.2<br>27 – 64	40.1 ± 8.8<br>27 – 65	0.111
LDL-cholesterol [mg/dl]	range (min –max)<br>median<br>quartile (25–75Q)	49 – 184<br>101<br>81 – 123	58 – 142<br>99<br>82 – 115	0.314*
Triglycerides [mg/dl]	mean ± SD<br>range (min-max)	119.2 ± 57.8<br>52 – 369	131.8 ± 70.1<br>39 – 469	0.312
eGFR [ml/min/1.73 m2]	mean ± SD<br>range (min-max)	152.9 ± 25.4<br>109.2 – 235.0	157.2 ± 26.3<br>117 – 223	0.398

* analysis with the non-parametric Mann-Whitney U test.

Table S2. Serum E-selectin, AGEs, MG, hsCRP, sAF and SDS PWV according to the SDS BMI values.

		**2 ≤ SDS BMI ≤ 4**	**SDS BMI > 4**	**p**
E-selectin [ng/ml]	mean ± SD<br>range (min-max)	59.2 ± 6.8<br>41.8 – 71.8	59.6 ± 7.2<br>43 – 75.3	0.786
AGEs [ng/ml]	mean ± SD<br>range (min-max)	46.3 ± 1.9<br>39.2 – 49.3	46.3 ± 1.7<br>41.1 – 49.6	0.944
MG [ng/ml]	range (min –max)<br>median<br>quartile (25–75Q)	167.2 – 291<br>227.2<br>211.9 – 258.8	156.4 – 297.8<br>224.4<br>208.2 – 237.6	0.579*
hs-CRP [μg/ml]	mean ± SD<br>range (min-max)	3.17 ± 0.41<br>2.36 – 4.08	3.31 ± 0.45<br>2.55 – 4.64	0.103
sAF [AU]	mean ± SD<br>range (min-max)	1.16 ± 0.24<br>0.53 – 1.73	1.27 ± 0.3<br>0.83 – 2.52	0.061
SDS PWV	mean ± SD<br>range (min-max)	–0.425 ± 1.91<br>(–9.43) – 2.33	–0.874 ± 2.117<br>(–7.937) – 1.34	0.371

* analysis with the non-parametric Mann-Whitney U test.

Table S3. Lipid and carbohydrate metabolism disorders in the studied population

Metabolicabnormality	N	%	Age range (years)	Median age	IQR (25–75%)	Sex ratio (F/M)	Clinical cut-off used*
Any metabolic abnormality	92	73.6	8.3 – 17.9	13.9	11.6 – 16.3	44 / 48	≥1 abnormality present
Total cholesterol ↑	17	13.6	9.3 – 17.7	14.3	11.1 – 16.5	6 / 11	>200 mg/dL
HDL cholesterol ↓	53	42.4	8.7 – 17.8	14.0	12.1 – 16.5	25 / 28	<40 mg/dL
Triglycerides ↑	38	30.4	8.7 – 17.8	14.3	12.1 – 16.3	16 / 22	>150 mg/dL
TG/HDL-C ratio ↑	76	60.8	8.7 – 17.8	13.9	11.9 – 16.3	37 / 39	>3.0
HOMA-IR ↑	34	27.2	9.2 – 17.4	13.3	11.6 – 15.2	35 / 32	>3.16 (<12 y) or >4.4 (≥12 y)

Table S4. The results of nine multiple regression of E-selectine, hsCRP and SD PWV height on AGEs, MG and sAF and covariates.

Explained variable	Main predictor name	Main predictor coeff. and p-value	Covariate coeff. and p-value				Overall p-value	Model
			Sex (female)	Age	Height (centile)	BMI (centile)		
E selectine	**AGEs**	0.55±0.36<br>0.13	0.61±1.25<br>0.63	-0.08±0.23<br>0.72	-0.01±0.02<br>0.61	0.06±0.19<br>0.74	0.64	OLS
	**MG**	0.01±0.03<br>0.70	-0.35±2.21<br>0.88	0.22±0.44<br>0.63	-0.02±0.04<br>0.57	-0.01±0.53<br>0.99	0.96	OLS
	**sAF**	-0.69±2.73<br>0.80	1.12±1.55<br>0.47	0.02±0.27<br>0.94	-0.01±0.02<br>0.59	0.09±0.22<br>0.69	0.95	OLS
hsCRP	**AGEs**	0.00±0.02<br>0.97	0.01±0.08<br>0.92	-0.01±0.02<br>0.56	0.00±0.00<br>0.61	-0.01±0.01<br>0.54	0.98	OLS
	**MG**	0.00±0.00<br>0.54	0.06±0.15<br>0.72	0.00±0.04<br>0.90	0.00±0.00<br>0.69	0.02±0.03<br>0.51	0.65	Q
	**sAF**	0.27±0.17<br>0.12	-0.01±0.10<br>0.96	-0.01±0.02<br>0.67	0.00±0.00<br>0.62	-0.01±0.01<br>0.59	0.68	OLS
SD PWV height	**AGEs**	-0.05±0.17<br>0.76	-0.45±0.51<br>0.39	0.01±0.08<br>0.92	0.00±0.01<br>0.98	0.05±0.15<br>0.75	0.58	Q
	**MG**	0.01±0.02<br>0.46	-0.25±1.15<br>0.83	-0.10±0.18<br>0.57	0.00±0.02<br>0.98	0.07±0.25<br>0.79	0.82	Q
	**sAF**	-0.77±0.83<br>0.36	-0.30±0.55<br>0.59	-0.03±0.08<br>0.70	0.00±0.01<br>0.87	0.06±0.15<br>0.67	0.80	Q

Explanation: The results include coefficients±SE in a top row , and coefficient p-values in a bottom row of a given regression. OLS – the ordinary least squares regression, Q – quantile regression (for median values).
